# Synergy of Photocatalysis and Adsorption for Simultaneous Removal of Hexavalent Chromium and Methylene Blue by g-C_3_N_4_/BiFeO_3_/Carbon Nanotubes Ternary Composites

**DOI:** 10.3390/ijerph16173219

**Published:** 2019-09-03

**Authors:** Huiwen Huo, Xinjiang Hu, Hui Wang, Jiang Li, Guangyu Xie, Xiaofei Tan, Qi Jin, Daixi Zhou, Chuang Li, Guoqiang Qiu, Yunguo Liu

**Affiliations:** 1 College of Environmental Science and Engineering, Central South University of Forestry and Technology, Changsha 410004, China; 2 Faculty of Life Science and Technology, Central South University of Forestry and Technology, Changsha 410004, China; 3 School of Architecture and Art, Central South University, Changsha 410082, China; 4 College of Environmental Science and Engineering, Hunan University, Changsha 410082, China; 5 Key Laboratory of Environmental Biology and Pollution Control (Hunan University), Ministry of Education, Changsha 410082, China

**Keywords:** hexavalent chromium, methylene blue, adsorption, photocatalysis, synergistic treatment

## Abstract

A novel graphite-phase carbon nitride (g-C_3_N_4_)/bismuth ferrite (BiFeO_3_)/carbon nanotubes (CNTs) ternary magnetic composite (CNBT) was prepared by a hydrothermal synthesis. Using this material, Cr(VI) and methylene blue (MB) were removed from wastewater through synergistic adsorption and photocatalysis. The effects of pH, time, and pollutant concentration on the photocatalytic performance of CNBT, as well as possible interactions between Cr(VI) and MB species were analyzed. The obtained results showed that CNTs could effectively reduce the recombination rate of electron-hole pairs during the photocatalytic reaction of the g-C_3_N_4_/BiFeO_3_ composite, thereby improving its photocatalytic performance, while the presence of MB increased the reduction rate of Cr(VI). After 5 h of the simultaneous adsorption and photocatalysis by CNBT, the removal rates of Cr(VI) and MB were 93% and 98%, respectively. This study provides a new theoretical basis and technical guidance for the combined application of photocatalysis and adsorption in the treatment of wastewaters containing mixed pollutants.

## 1. Introduction

With the development of many industrial technologies, pollutants discharged into the environment have become increasingly toxic and chemically complex [1]. Generally, industrial wastewaters tend to contain both organic pollutants and heavy metals, and their proper treatment is a very challenging task because it requires the use of special techniques and reaction conditions [2]. Chromium has two valence states in water, Cr(VI) and Cr(III), among which Cr(VI) is highly toxic, water-soluble, mobile, and easy to accumulate. It is allergenic, carcinogenic, teratogenic, and mutagenic as it could interfere with the transcription process of DNA [3], therefore Cr(VI) is classified as a surface water pollutant of the first type. Comparatively, Cr(III) is less toxic and can be easily removed by forming the Cr(OH)_3_ precipitate under alkaline conditions. Methylene blue (MB) is a commonly used cationic dye that is chemically stable and can affect the growth of aquatic plants and microorganisms, thereby causing serious ecological problems [4,5]. Cr(VI) and MB simultaneously exist in wastewaters produced during electroplating, printing, and dyeing as well in the leather, metallurgical, cosmetics, and pigments industries [6]. Therefore, the effective treatment of wastewater aimed at the removal of heavy metals and organic pollutants has become the focus of many studies in the field of environmental chemistry [7].

In recent years, various methods for treating such mixed wastewaters have been developed including photocatalysis, adsorption, ion exchange, membrane filtration, chemical precipitation, flocculation, oxidation, and aerobic/anaerobic biological treatment (for low and high concentration organic wastewater, respectively) [8,9]. The advantages of the first two techniques over other methods include their simplicity, lower costs, and higher treatment and Cr(VI) decontamination efficiencies [10]. These technologies are widely used in the industry, and proper selections of the catalyst and adsorbent are the main factor affecting their performance [11,12].

Graphite-phase carbon nitride (g-C_3_N_4_) is a typical nontoxic metal-free semiconductor [10]. It consists of only two elements, C and N, which are abundant in the earth core and can be chemically modified through simple inexpensive routes [13]. g-C_3_N_4_ is an allotrope of carbon nitride with a stacked two-dimensional structure and band gap of ca. 2.7 eV, which suggests its possible application in sunlight harvesting as well as high chemical and thermal stabilities [14]. The C and N atoms of g-C_3_N_4_ are sp^2^-hybridized to form highly delocalized conjugated π-bonds [15]. g-C_3_N_4_ has been widely used in the photocatalytic reduction of heavy metals [16], the photocatalysis hydrogen production [17], and photodegradation of organic pollutants [18]. However, bulk g-C_3_N_4_ has a small specific surface area, very few active sites, and a high electron-hole recombination rate, which do not allow significant enhancement of its photocatalytic performance [19]. Hence, various researches have tried to improve the photocatalytic activity of this material by coupling [20] and surface modification [21].

Bismuth ferrite (BiFeO_3_) is a photocatalytic material with a wide range of potential applications, which possesses a narrow band gap and is responsive to visible light [22]. Furthermore, it can simultaneously exhibit ferroelectric and magnetic properties at room temperature [21]. Combing g-C_3_N_4_ with BiFeO_3_ to produce the g-C_3_N_4_/BiFeO_3_ (CNB) composites may reduce the recombination rate of photogenerated electron-hole pairs and expand the photo-response range, while the strong magnetic properties of BiFeO_3_ can be used in the solid-liquid separation by a magnet [23]. In addition, under visible light radiation, BiFeO_3_ generates photoexcited electrons that react with H_2_O to form **·**OH, highly reactive free radicals, that can effectively degrade organic pollutants such as MB [24]. However, we also found that the photoreduction rate of Cr(VI) by CNB was not very high, indicating that although the coupling of g-C_3_N_4_ and BiFeO_3_ could improve the photocatalytic efficiencies of these compounds to some extent, the efficiency of the electron transfer between them remained limited.

Carbon nanotubes (CNTs) are nanomaterials with a large specific surface area, good electrical conductivity, and low solubility that are widely utilized in adsorption treatment processes [25]. The p-electrons of their C atoms form a large range of delocalized π-bonds with a strong conjugation effect, which promotes the storage and transfer of photoexcited electrons [26]. Therefore, combining g-C_3_N_4_, BiFeO_3_, and CNTs to form a ternary magnetic composite may not only increase the specific surface area and number of active sites [27], but also reduce the recombination rate of photogenerated carriers, thus improving the photocatalytic properties of the resulting material.

Although many research groups have investigated various technologies for the treatment of solutions containing the Cr(VI) or MB species, very few of them discussed their simultaneous removal through synergistic photocatalysis and adsorption. Therefore, in this study, g-C_3_N_4_, BiFeO_3_, and CNTs were combined to produce a ternary magnetic composite CNBT, and its synergistic adsorption and photocatalytic properties towards the removal of Cr(VI) and MB from wastewaters were explored. Additionally, the influences of the solution pH, pollutant concentration, and reaction time on the adsorption and photocatalytic processes were determined, and possible mechanisms of the adsorption and photocatalytic reactions were suggested.

## 2. Materials and Methods

### 2.1. Preparation of Functionalized Ternary Magnetic Composite CNBT

Fe(NO_3_)_3_, Bi(NO_3_)_3_, K_2_Cr_2_O_7_, melamine, methyl alcohol, ethyl alcohol, ethylene glycol monomethyl ether, and methyl ether were purchased from China National Pharmaceutical Group Chemical Reagent Co. Ltd. (Shanghai, China). Citric acid was supplied by Shanghai Mclean Biochemical Technology Co. Ltd. (Shanghai, China). The chemicals were of the analytical grade. CNTs (Muti Wall Carbon nanotubes, Main Range of Diameter: 10–20 nm, purity > 97%) was purchased from Shenzhen Nanotech Port Co. Ltd. (Shenzhen, China).

First, 20.0 g of melamine was washed by distilled water and absolute ethanol in a crucible. The supernatant was removed, and the obtained precipitate was placed into a muffle furnace for dehydration at 70 °C in air for 20 min without capping. Subsequently, the crucible was covered and calcined at 600 °C for 3 h in air. After cooling at ca. 25 °C, the precipitate was ground and sieved (#100 mesh size) to obtain g-C_3_N_4_.

BiFeO_3_ was prepared by a sol-gel method [26]. First, 0.08 mol of Fe(NO_3_)_3_ and 0.08 mol of Bi(NO_3_)_3_ were dissolved in 200 mL of ethylene glycol methyl ether followed by the addition of 0.2 mL of nitric acid (0.1 mol/L) obtained a mixed solution. Next, 0.08 mol of citric acid was dissolved in 100 mL of ethylene glycol and added to the above mixed solution. The obtained mixture was stirred at 60 °C for 1 h and then heated to 100 °C for 17 h in air. The resulting yellow gel was transferred to a crucible and heated first to 200 °C for 1 h and then to 500 °C for 2 h in a muffle furnace in air. After cooling at ca. 25 °C, the produced solid was ground to obtain the BiFeO_3_ powder.

CNB and CNBT were synthesized by ultrasonically dispersing their components in methanol at specified ratios for 3 h followed by dehydration. The mass ratio of g-C_3_N_4_ to BiFeO_3_ for CNB was 1:1, and that of g-C_3_N_4_, BiFeO_3_, and CNTs for CNBT was 3:3:1.

### 2.2. Characterization

The material microstructure was characterized by a QUANTA250 field emission scanning electron microscope (FE-SEM; FEI, Hillsboro, OR, USA), the samples were directly attached to the conductive adhesive, then were tested with the ETD morphology mode under a 20 KV working voltage and 0° angle after gold spraying. Transmission electron microscope (TEM) analyses were applied a Tecnai G2 F20 (TEM; FEI, Hillsboro, OR, USA). The samples were analyzed by a NICOLET 5700 Fourier transform infrared (FTIR) spectrometer (Thermo Nicolet Corporation, Madison, WI, USA) under the acquisition range of 400–4000 cm^−1^ (number of scans 64, resolution 4 cm^−1^). Thermogravimetric (TG) and differential scanning calorimetry (DSC) curves were recorded in air from room temperature to 1000 °C using an SDT Q600 synchronous thermal analyzer (New Castle, TA, USA) at a flow rate of 100 mL/min and heating rate of 10 °C/min. The Brunner–Emmet–Teller (BET) specific surface areas of the studied materials were determined using an ASAP2460 4MP instrument (Norcross, GE, USA). The magnetic properties of the samples were evaluated by a MPMS-XL-7 vibrating sample magnetometer (VSM; Quantum Design Instruments, O’Fallon, MO, USA). The electron spin resonance (ESR) signals produced by spin-trapped radicals were examined on a JES FA200 spectrometer (JEOL, Tokyo, Japan) using the spin-trapping reagent 5,5-dimethyl-1-pyrroline N-oxide (DMPO) under visible light irradiation. Phase compositions of the materials were analyzed by the X-ray diffraction (XRD) on a D/max-2500 (Rigaku, Tokyo, Japan) diffractometer with a Cu Kα radiation, while their elemental compositions were determined by the X-ray photoelectron spectroscopy (XPS, ESCALAB 250XI, Thermo Fisher Scientific, Waltham, MA, USA). The XPS data acquisition was under the standard lens mode, Al Ka source type (hv = 1486.6 eV), 30.0 eV pass energy, 500 μm spot size, C1s 284.8 eV as the reference point, and the energy step size of the full XPS spectra and the characteristic spectra were 1.0 eV and 0.5 eV, respectively. The photoluminescence (PL) spectra were recorded on a FLS 980 fluorescence spectrophotometer (Edinburgh Instruments, Livingston, UK). Ultraviolet (UV)-visible diffuse reflectance spectroscopy (DRS) analyses of the samples were conducted by a diffuse reflectance spectrophotometer (UH4150 UV-vis, Hitachi, Tokyo, Japan) using BaSO_4_ as a reference under the condition of start wavelength 800 nm, end wavelength 200 nm, scan speed 300 nm/min, lamp change wavelength 350 nm, path length 10 mm, step size 0.50 nm.

### 2.3. Photoelectrochemical Measurements

The photocurrent responses of the studied materials were measured using a photochemical workstation (CHI 660E) with a conventional three-electrode model [28]. g-C_3_N_4_, BiFeO_3_, CNTs, CNB, and CNBT samples were examined by a current-time method, in which the Pt and Ag/AgCl electrodes served as the counter and reference electrodes, respectively. The working electrode was fabricated as follows. First, 20 mg of the sample was suspended in a 2 mL nafion (0.25 vol%) to form a slurry, which was fully dispersed by ultrasonication for more than 2 h. Next, 0.2 mL of the dispersed slurry was uniformly dropped onto the conductive side of the clean FTO glass sheet at 80 °C, then used as a working electrode [5]. To obtain a working area of 1 × 1 cm^2^, the extra parts of the slurry were scraped off. The electrolyte of the three-electrode system consisted of a 0.5 mol/L Na_2_SO_4_ solution. The light source was a xenon lamp with a power of 300 W (CEL-HXF300, Beijing China Education Au-light Co. Ltd., Beijing, China) containing a UV filter (λ > 400 nm). The distance between the light source and the surface of the working electrode was set to 15 cm. In addition, the light and dark states were alternated in 20-s intervals during the test.

### 2.4. Photocatalytic Activity Testing

To prepare the samples for photocatalytic activity testing, 2.829 g of potassium dichromate was heated in an oven to 110 °C for 2 h, after which the annealed powder and 1 g of MB were dissolved separately in ultrapure water inside 1000-mL volumetric flasks to obtain 1 g/L Cr(VI) and MB stock solutions, respectively. Different concentrations of the Cr(VI) and MB solutions used in the experiments were achieved by diluting their corresponding stock solutions.

The schematic diagram of the photocatalytic activity test reaction scheme is shown in Figure S3. A 300-W xenon lamp (CEL-HXF300, Beijing China Education Au-light Co. Ltd.) with an ultraviolet filter (λ > 400 nm) was used as a visible light source. Multiple 200-mL mixed solutions containing 5 mg/L of Cr(VI) and 20 mg/L of MB were prepared, and their pH values were adjusted using 0.1 or 1 mol/L HCl and NaOH solutions followed by the addition of 0.5 g of the catalyst. Each solution was first stirred magnetically at a speed of 1000 rpm under dark conditions for 1 h to reach the adsorption-desorption equilibrium between the contaminant and the photocatalyst, and then radiated with visible light for 4 h. The xenon lamp was equipped with an ultraviolet filter, and the beam was directed to the lens to shine vertically on the liquid surface, with a vertical distance of about 15 cm. The solution was multiplied in the 500 mL beaker and placed on a magnetic stirrer at a speed of 1000 rmp for 4 h. In the process, ca. 5 mL of the treated solution was passed through a 0.45-μm filter (Hydrophilic, MCE) to filtration every 30 min. The concentrations of the Cr(VI) and MB species left in the reacted solutions were measured by a UV-visible spectrophotometer (UV2600, UNICO, Shanghai, China) at wavelengths of 540 nm and 665 nm, respectively. To ensure the accuracy of the experimental results, each group of experiments was conducted in triplicate.

### 2.5. Adsorption Studies

After adjusting the pH of the mixed solutions of Cr(VI) and MB with HCl and NaOH, 0.05 g of the composite was added to the conical flasks containing 20 mL of these solutions. The flasks were placed in a rotary shaker and agitated at a constant temperature of 25 °C and speed of 150 rpm for 5 h. After the reaction, 5 mL of each solution was passed through a 0.45-μm filter, and the remaining concentrations of Cr(VI) and MB were measured by a colorimetric method at wavelengths of 540 nm and 665 nm, respectively, using the UV-visible spectrophotometer. Each group of experiments was done in triplicate. In addition, the effects of different materials, pH value (2.0–11.0), initial concentrations of the Cr(VI) (1–50 mg/L) and MB (5–200 mg/L) species, and reaction time (5–300 min) on the adsorption capacity were studied. The adsorbed amount was calculated by the following equation:

(1) $$q_{e}\;=\;\frac{{(C}_{0}-C_{e})V}{W}$$

where *q*_e_ represents the amount of the adsorbed material at equilibrium (mg/g); *C*_0_ and *C*_e_ are the initial and equilibrium concentrations of Cr(VI) or MB in the solution (mg/L), respectively, *V* is the solution volume (L), and W indicates the amount of the added material (g) [29]. The adsorption models applicable under different control conditions are described in the Supplementary Material section. The different model parameters are listed in Tables S1 and S2, the fitted models are shown in Figures S1 and S2, respectively.

## 3. Results and Discussion

### 3.1. Sample Characterization

#### 3.1.1. Electron Microscopy

**Figure 1:**
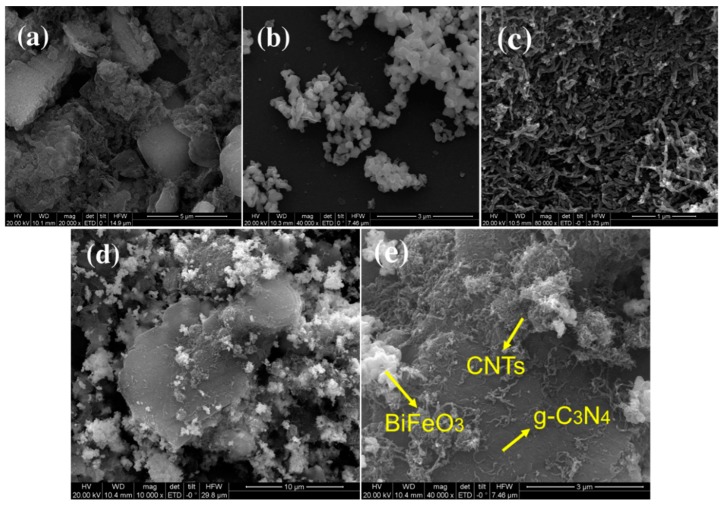
SEM images of (**a**) graphite-phase carbon nitride (g-C_3_N_4_), (**b**) bismuth ferrite (BiFeO_3_), (**c**) carbon nanotubes (CNTs), (**d**) g-C_3_N_4_/BiFeO_3_ (CNB), and (**e**) carbon nanotubes ternary magnetic composite (CNBT).

The morphologies and structures of g-C_3_N_4_, BiFeO_3_, CNTs, CNB, and CNBT were analyzed by the scanning electron microscopy (see Figure 1). The g-C_3_N_4_ sample depicted in Figure 1a exhibits many irregular small crystals, which are closely packed in layers. As shown in Figure 1b, BiFeO_3_ represents a fine-grained crystal with a size of about 100 nm, and CNTs are a dense tubular material as shown in Figure 1c. In the CNB and CNBT composites depicted in Figure 1d,e, respectively, the surface of the original granular BiFeO_3_ was covered with a number of CNTs, which reduced the sharpness of BiFeO_3_ and increased the specific surface area due to the combination of CNTs and g-C_3_N_4_; g-C_3_N_4_ has a lamellar shape with a flat surface and randomly dispersed BiFeO_3_ particles; and CNTs have large aspect ratios and are loosely coupled to the other two components.

**Figure 2:**
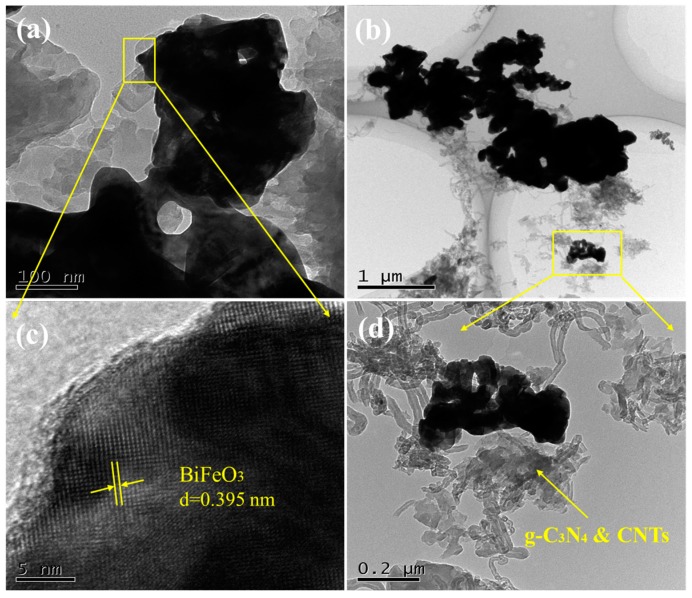
TEM images of (**a**,**c**) CNB, (**b**,**d**) CNBT.

The CNB and CNBT samples were also analyzed by the transmission electron microscopy (the obtained results are shown in Figure 2). Figure 2a,c characterize the microscopic morphology of the CNB material. Both its g-C_3_N_4_ and BiFeO_3_ components are irregularly block-shaped and tightly bound, while the color of BiFeO_3_ is relatively dark. The resolution of the crystal plane is ca. 0.395 nm, which is similar to the results of the literature studies [30,31]. Figure 2b,d show that the coupling of linear CNTs with g-C_3_N_4_ produces the composite with a large specific surface area, while the morphology of CNTs does not significantly change.

#### 3.1.2. FTIR

**Figure 3:**
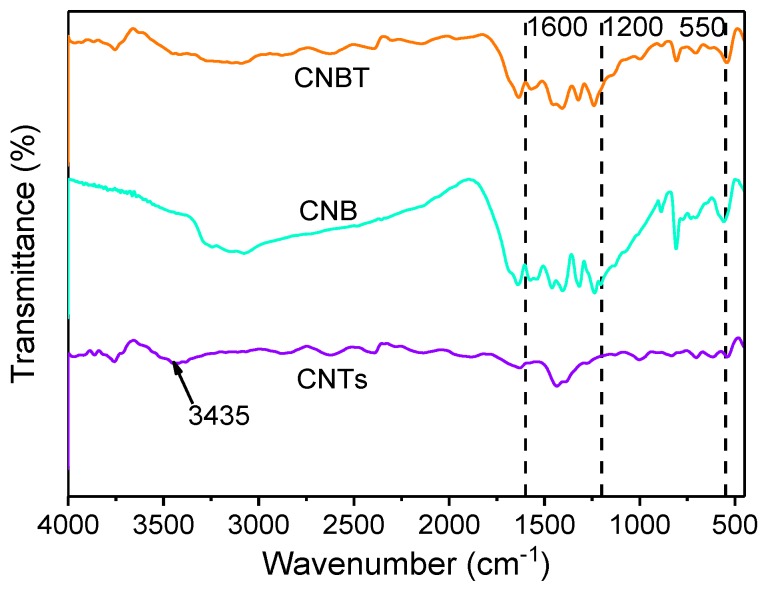
FTIR spectra of CNTs, CNB and CNBT.

The FTIR spectral characteristics of g-C_3_N_4_ and BiFeO_3_ were determined in previous studies [26]. The absorption band of g-C_3_N_4_, was located close to 1639 cm^−1^ due to the C=N stretching vibration [32], and its absorption peaks were centered at around 808 cm^−1^ and 1200–1600 cm^−1^, representing the out-of-plane bending modes of C-N heterocycles and the typical aromatic C-N stretching vibrations, respectively [26]. The absorption peak of BiFeO_3_ was centered near 550 cm^−1^ [26]. The FTIR spectra of the CNB and CNBT composites and CNTs are displayed in Figure 3. For CNTs, the absorption peak was weaker than those of the other materials and centered at a frequency of 3435 cm^−1^ characteristic of the O-H bonds, which might be due to the adsorption of water molecules on the nanotube surface [9,33]. The broadband spanning from 1000 to 1400 cm^−1^ corresponded to the C=O bond stretching vibrations [34]. The FTIR peak of the CNBT composite centered at 1650–1709 cm^−1^ was weaker than that of CNB, owing to the presence of CNTs. In addition, the CNBT peaks were compared with the characteristic peaks of the g-C_3_N_4_, BiFeO_3_, and CNTs individual components; the obtained results revealed that the basic structure of each material was not destroyed during the compounding process.

#### 3.1.3. TG-DSC

Figure S4 shows that both g-C_3_N_4_ and BiFeO_3_ exhibit a weak endothermic DSC peak at 60 °C because of the volatilization of the adsorbed water. However, no obvious weight loss was detected on the corresponding TG curve, indicating that the analyzed sample contained a very little amount of water. The mass loss of g-C_3_N_4_ was about 90% at 550–705 °C, and the corresponding DSC curve had an exothermic peak at 703 °C caused by structural damage. In addition, BiFeO_3_ produced no other apparent endothermic or exothermic peaks throughout the heating process and did not lose any weight. CNTs lost about 92% of their original weight in the temperature region of 560–660 °C. Strong and weak exothermic peaks were observed at 607 °C and 635 °C, which might be due to the oxidative decomposition of the surface groups and oxidative combustion of the materials [9]. The CNBT composite exhibited a strong exothermic peak at 527 °C, and the mass loss at 480–580 °C was about 52% (the further increase in temperature did not reduce its mass significantly).

#### 3.1.4. VSM Measurements

The magnetization hysteresis loops of BiFeO_3_, CNB, and CNBT were recorded by vibrating a sample magnetometry to determine their magnetic properties. Figure S5 illustrates the data listed in Table S3, shows the magnetic properties of BiFeO_3_, CNB, and CNBT, the magnetic retention of all three materials was weak (0.29, 0.14, and 0.12 emu/g for BiFeO_3_, CNB, and CNBT, respectively), and the magnetization phenomenon almost disappeared in the absence of an applied magnetic field, suggesting that the samples exhibit a superparamagnetic behavior at room temperature, and their hysteresis loops were sigmoidal closed curves [35]. In addition, the analysis shows that BiFeO_3_ is a stronger superparamagnetic than CNB and CNBT, while CNBT has the weakest magnetic properties. Therefore, after the wastewater treatment, the separation and recycling of the material can be realized by applying a magnetic field, thereby simplifying the treatment process.

#### 3.1.5. ESR

**Figure 4:**
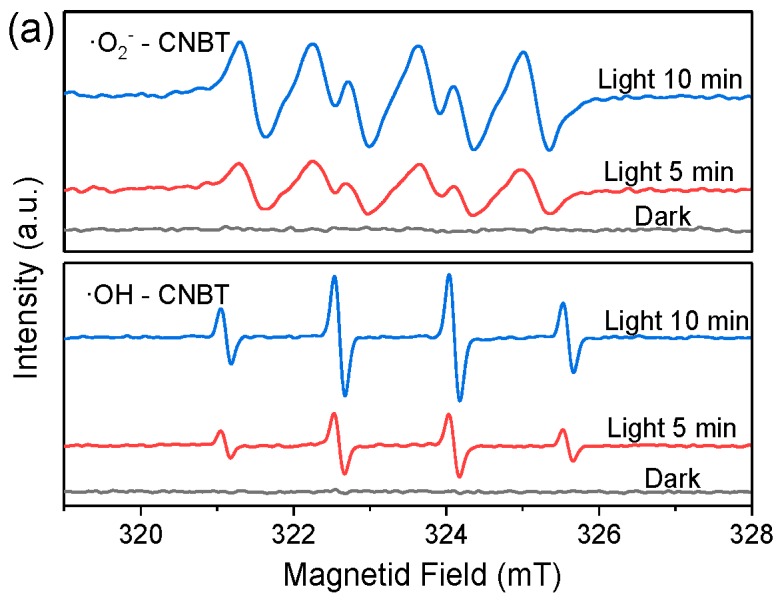

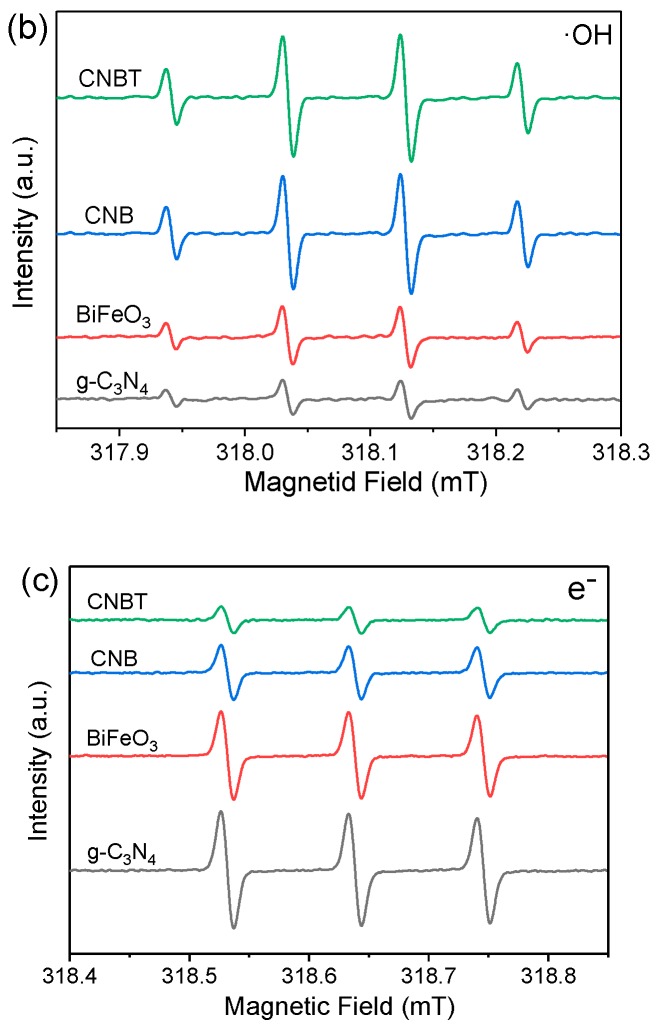
(**a**) ESR spectra of radical adducts trapped by ·O_2_^−^ and ·OH for CNBT in the dark and under visible light irradiation. (**b**,**c**) ESR spectra of g-C_3_N_4_, BiFeO_3_, CNB, and CNBT by ·OH (**b**) and e^−^ (**c**) exposed to visible light for 10 min.

Since the **·**OH, e^−^, and **·**O_2_^−^ species are important intermediates formed during the photocatalytic process [36], the synthesized material was analyzed by the ESR, and the amounts of both the free radicals and e^−^ were determined from the detected signal strengths. Figure 4a displays the ESR curves of **·**OH and **·**O_2_^−^ produced by the CNBT composite. It shows that the number of the **·**OH and **·**O_2_^−^ species generated in the dark is very small, and their corresponding signals are also very weak. After the light was applied for 5 min, the number of the two free radicals increased significantly. When the light was applied for 10 min, the number of these free radicals almost doubled as compared with that obtained after 5 min of illumination. These results revealed that the CNBT composites could be excited to generate a large amount of the **·**OH and **·**O_2_^−^ species under more permanent light conditions. Figure 4b,c show the ESR spectra of **·**OH and e^−^ recorded for g-C_3_N_4_, BiFeO_3_, CNB, and CNBT after 10 min of illumination. The higher was the test signal intensity, the greater was the ability of a material to produce the **·**OH radicals. However, the opposite trend was observed for the e^−^ species (the higher the intensity, the smaller the number of e^−^). From the signal intensity curves depicted in Figure 4b,c, it can be concluded that the abilities of different materials to generate **·**OH and e^−^ may be ranked as follows: CNBT > CNB > BiFeO_3_ > g-C_3_N_4_, indicating that the CNBT composite has the strongest **·**OH and electron generation capabilities.

#### 3.1.6. XRD

**Figure 5:**
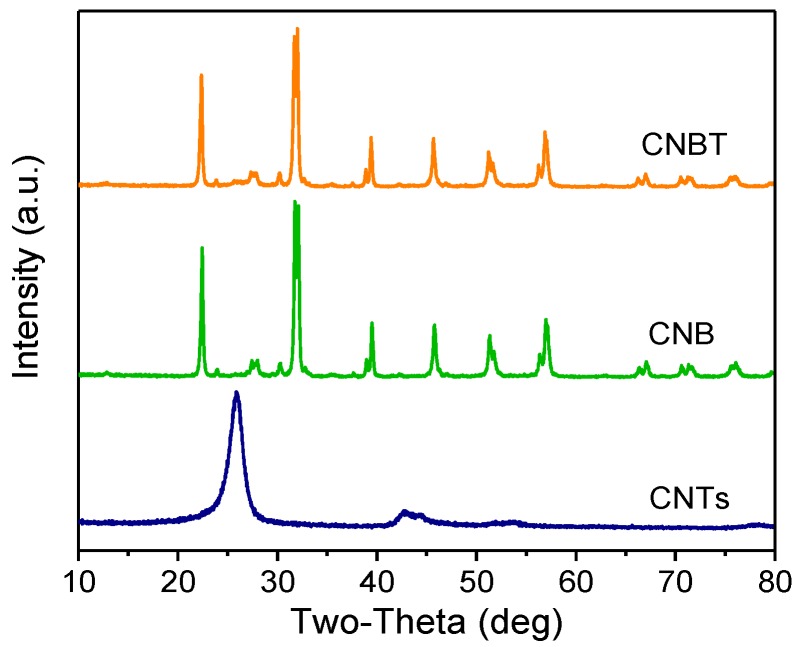
XRD patterns of CNTs, CNB, and CNBT.

The XRD patterns recorded for the CNTs, CNB, and CNBT are shown in Figure 5. In our previous study, the XRD patterns of the g-C_3_N_4_ and BiFeO_3_ individual components were analyzed [26]. The g-C_3_N_4_ peaks were centered at 2θ = 12.9° and 27.6°, while the characteristic peaks of BiFeO_3_ formed a diffraction pattern containing the (012), (104), (110), (006), (202), (024), (116), (122), (018), (202), (214), (300), (208), and (220) reflections of the planar BiFeO_3_ single-phase perovskite structure. The results presented in Figure 5 show that the CNTs produced two different diffraction peaks centered at 2θ = 25.9° and 42.8°, which corresponded to the (002) and (101) crystal planes, respectively (JCPDS 01-0646; note that the intensity of the latter peak was relatively small) [37]. The XRD patterns of CNB and CNBT contain the characteristic peaks of g-C_3_N_4_, BiFeO_3_, and CNTs. However, the diffraction peak of the CNTs is very weak, which also confirms that the content of CNTs in the composite is smaller than those of the g-C_3_N_4_ and BiFeO_3_ components. At the same time, no new peaks were detected in the diffraction patterns of all materials, indicating that no impurities were introduced during the material preparation and that the purity of the composite was relatively high.

#### 3.1.7. XPS

**Figure 6:**
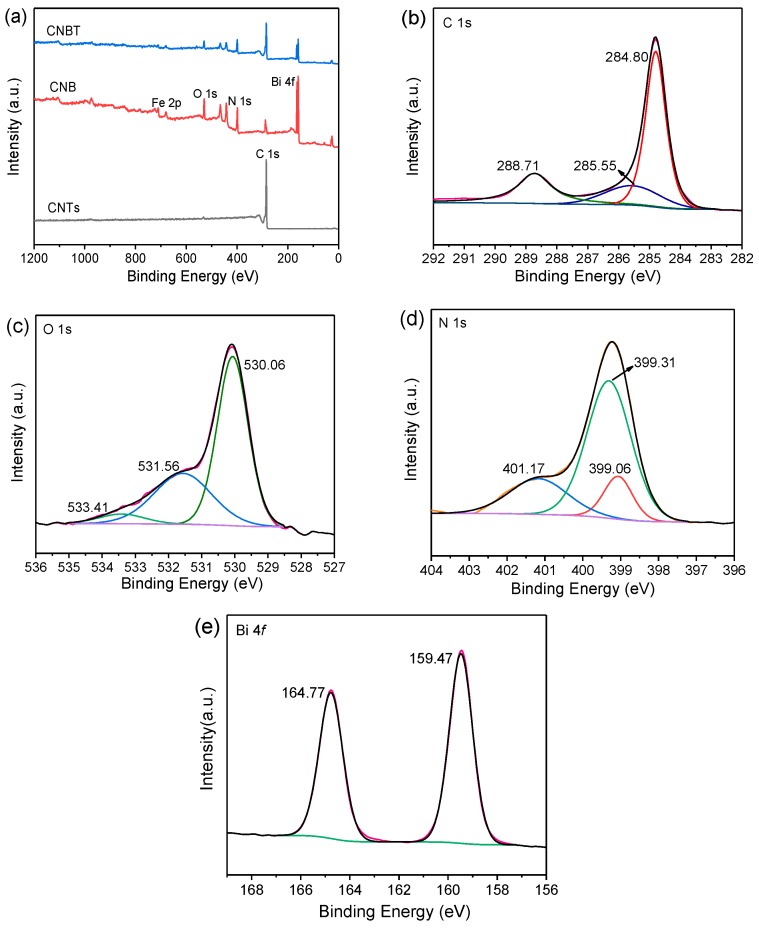
(**a**) Full XPS spectra of CNTs, CNB, and CNBT. Characteristic spectra of CNBT, (**b**) C 1s, (**c**) O 1s, (**d**) N 1s, (**e**) Bi 4f.

In order to determine the chemical compositions of the CNBT composite and possible interaction between its various components, the XPS analysis was performed on the CNTs, CNB, and CNBT materials (the full XPS spectra of them are shown in Figure 6a). The characteristic peaks of the individual components had different degrees of correspondence in the composite. Figure 6b contains the C 1s XPS spectrum of the CNBT, which was fitted with three different Gaussian peaks centered at 284.80, 285.55, and 288.71 eV. The first two peaks corresponded to either the sp^2^ hybrid N−C=N or C−C structure and sp^3^ hybrid structure of C−N_3_ in g-C_3_N_4_, respectively [38]. The peak at 288.71 eV was attributed to the O−C=O structure in the constituent CNTs [39,40]. In Figure 6c, the characteristic peaks of O 1s centered at 530.06, 531.56, and 533.41 eV were attributed to the presence of Bi−O (BiFeO_3_), C=O, or O−C=O, and the hydroxyl O−H (CNTs) species, respectively [26,41]. In Figure 6d, the N−C_3_ tertiary nitrogen structure and sp^2^ C−N=C hybrid structure formed by the N atom as well as the C_2_−N−H structure formed by the attachment of the amide N-containing group to the edge of the layer with binding energies of 399.31, 399.06, and 401.17 eV, respectively [36,38]. As shown in Figure 6e, the XPS spectrum of Bi can be distinguished by the presence of the 4 *f*_7/2_ and 4 *f*_5/2_ Gaussian peaks with binding energies of 159.47 eV and 164.77 eV, respectively, which correspond to the spin-orbit of Bi [31]. From these results, it can be concluded that the Bi element existed in the form of Bi^3+^ oxide in the produced composite [42].

### 3.2. Investigation on Charge Separation and Optical Properties

**Figure 7:**
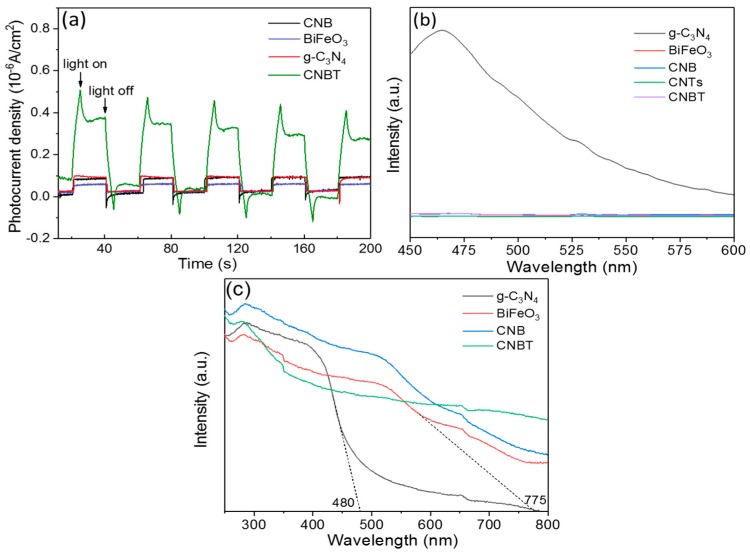
(**a**) Photocurrent response density. (**b**) Photoluminescence spectra. (**c**) UV-Vis spectra.

The results of the photocurrent test are depicted in Figure 7a. They show that g-C_3_N_4_, BiFeO_3_, CNB, and CNBT can produce electrons at the moment of illumination and generate an electric current due to the photocatalytic effect [26]. The photocurrent in CNBT was much stronger than those in the other materials, confirming that the former possessed a higher separation efficiency of photogenerated hole-electron pairs and larger electron conductivity.

The PL properties of the synthesized materials were examined to further determine the recombination rates of photoexcited electron-hole pairs [43]. Generally, the higher the fluorescence signal, the greater the recombination rate of such pairs [28]. In Figure 7b, the pure g-C_3_N_4_ exhibits the strongest fluorescent peak, owing to its highest recombination rates of photogenerated electron-hole pairs. After combining g-C_3_N_4_ with BiFeO_3_ and CNTs, the PL peak intensity was significantly reduced, which indicated that the addition of BiFeO_3_ and CNTs could inhibit the photoelectron-hole pair recombination, thereby improving the photocatalytic efficiency of the composite [38].

The optical properties of a material directly affect its light absorption capacity [44]. In this work, UV-visible diffuse reflection studies of g-C_3_N_4_, BiFeO_3_, CNB, and CNBT were performed to examine their light absorption characteristics. Figure 7c clearly shows that the edge of the light absorption region of g-C_3_N_4_ is ca. 480 nm, which is close to the absorption regions of g-C_3_N_4_ prepared in previous studies [45,46,47]. BiFeO_3_ had a relatively high visible light absorption capacity, and the edge of its absorption region was ca. 775 nm [48]. Figure 7c shows that CNB has a wider photo-response range than that of the pure g-C_3_N_4_ due to the addition of BiFeO_3_ and that the photo-response range of the CNBT composite is further broadened after adding the CNTs. The band gap energy was calculated by the following formula:

E_g_ = 1240/λ (2)

where λ is the wavelength (nm) of the absorption edge, and E_g_ is the band gap energy of the semiconductor (eV) [43,49]. This formula shows that the band gaps of g-C_3_N_4_ and BiFeO_3_ are 2.58 eV and 1.6 eV, respectively. According to Figure 7c, the band gaps of the studied materials can be ranked in the order of g-C_3_N_4_ > BiFeO_3_ > CNB > CNBT. The lowest band gap of CNBT can facilitate the excitation and transition of the electrons, thereby improving the photocatalytic performance of the material [38].

### 3.3. Adsorption Studies

Before conducting the photocatalytic performance tests, a series of adsorption experiments were performed to compare the adsorption properties of different materials. By taking into account the characteristics of wastewater, the influences of pH, and the concentrations of Cr(VI) and MB on their adsorption properties were analyzed.

#### 3.3.1. Adsorption Properties of Different Materials

Figure S6 shows the adsorption efficiencies of g-C_3_N_4_, BiFeO_3_, CNB, and CNBT in the mixed solution of MB and Cr(VI). After 5 h of reaction, the adsorption capacity of g-C_3_N_4_ towards MB was about 0.63 mg/g; however, no apparent adsorption of the Cr(VI) ions was observed. The adsorption capacities of BiFeO_3_ towards MB and Cr(VI) reached 4.85 mg/g and 0.78 mg/g, respectively. While the adsorption efficiency of CNB was lower than that of BiFeO_3_ due to the combination of g-C_3_N_4_ and BiFeO_3_ that likely blocked some adsorption sites on the BiFeO_3_ surface. Compared with CNB, CNBT has a higher specific surface area and more adsorption sites, which promoted the adsorptions of MB and Cr(VI) reaching the adsorption capacities of 5.33 mg/g and 1.04 mg/g, respectively. According to the results of the BET measurements, CNBT had the highest pore specific surface area of 38.3291 m^2^/g and a pore volume of 0.1576 cm^3^/g, which also contributed to its superior adsorption properties.

#### 3.3.2. Adsorption Efficiencies at Different pH

**Figure 8:**
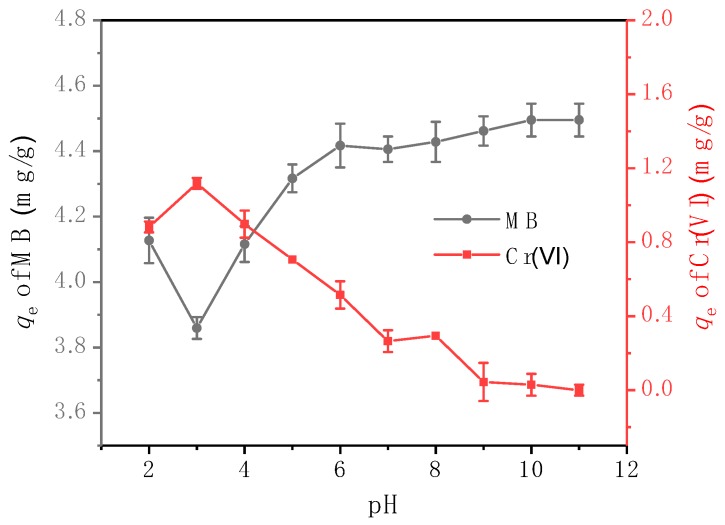
Effect of pH values on the adsorption of Cr(VI) and MB with CNBT: *C*_0Cr(VI)_ = 5 mg/L, *C*_0MB_ = 20 mg/L, m/V = 2.5 g/L, T = 25 °C.

The pH value of the solution is an important parameter of the adsorption process, which affects not only the surface charge of the adsorbent, but also the physicochemical properties of the adsorbate [7,34]. In this work, the pH values were adjusted to 2.0–11.0 using 0.1 and 1 mol/L of the NaOH and HCl solutions, and the adsorption reaction was performed for 5 h at 25 °C. According to the results presented in Figure 8, the adsorption efficiency strongly depends on the pH value. When the pH was above three, the adsorption of Cr(VI) was suppressed with an increase in pH, whereas the opposite trend was observed for MB because the higher pH values increased the number of negative charges on the CNBT surface due to protonation [50]. Cr(VI) exists mainly in the form of HCrO_4_^−^ under acidic conditions and in the form of Cr_2_O_7_^−^ in the alkaline solutions. Such Cr(VI) species with negative charges are electrostatically repelled from the negatively charged CNBT species. In contrast, the cationic dye MB is positively charged because it can ionize chloride ions and color groups in the solution and thus is attracted by the negative charges of CNBT, which increases the MB removal rate.

When the pH was between two and three, the actual adsorption effect of the composite on the two pollutants was opposite to the overall trend. This phenomenon can be explained by the fact that under strong acidic conditions, MB was protonated to become positively charged, whereas Cr(VI) possessed a negative charge in the form of HCrO_4_^−^. These two compounds could react to form a flocculent chelate complex, which reduced the amount of Cr(VI) adsorbed by the material. Therefore, in the case of a large MB concentration, the reduction in the number of the Cr(VI) adsorption sites provided more adsorption sites for MB. As a result, the material exhibited stronger MB adsorption properties. When the pH was between six and eight, the form of Cr(VI) changed dramatically, mainly reflected in the transformation of HCrO_4_^−^ to CrO_4_^2−^, which made the unstable trend for Cr(VI) a possibility [26].

### 3.4. Photocatalytic Experiments

#### 3.4.1. Photocatalytic Properties of Different Materials

**Figure 9:**
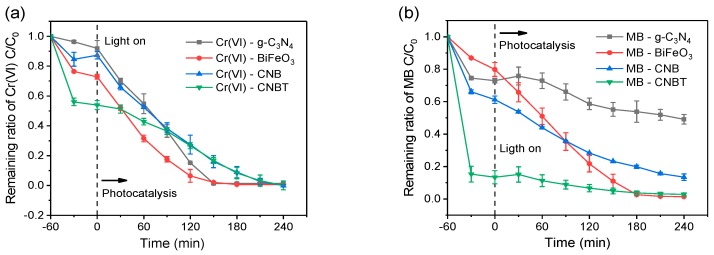
Effects of different kinds of materials on Cr(VI) (**a**) and MB (**b**) reduction by g-C_3_N_4_, BiFeO_3_, CNB, and CNBT under visible light irradiation when Cr(VI) mixed with MB: *C*_0Cr(VI)_ = 5 mg/L, *C*_0MB_ = 20 mg/L, m/V = 2.5 g/L, pH = 2.0. (−60–0 min: Adsorption process, 0–240 min: Photocatalysis process).

Figure 9 describes the removal of the Cr(VI) and MB species from the mixed wastewater by the four materials under the same reaction conditions. After 1 h of the treatment, the removal rates of Cr(VI) and MB by the CNBT composite were 50% and 75% within the first half an hour and 55% and 79% by the end of the first hour, respectively, indicating that the adsorption-desorption equilibrium was almost reached within the study period. After the application of light, the removal rates of both pollutants significantly increased. During the 4 h photocatalytic treatment, g-C_3_N_4_ and BiFeO_3_ exhibited different adsorption capacities toward Cr(VI) (see Figure 9a), but no significant differences in their photocatalytic abilities were observed. After the photocatalytic treatment, the removal rate of Cr(VI) exceeded 98%; however, g-C_3_N_4_ demonstrated a poor photocatalytic degradation of MB, as shown in Figure 9b. After the synergistic treatment involving both the adsorption and photocatalysis reactions, about 50% of MB was removed from the mixed solution, which might be caused by the relatively low number of the **·**OH radicals produced by the g-C_3_N_4_-containing photocatalytic systems, resulting in the undesirable photodegradation of MB. In addition, CNBT exhibited a stronger photocatalytic effect than that of CNB because the added CNTs played the role of channels that facilitated the electron transfer process. As a result, the number of active sites increased due to the large surface area, which increased the reaction efficiency.

#### 3.4.2. The Relationship between the Two Pollutants

**Figure 10:**
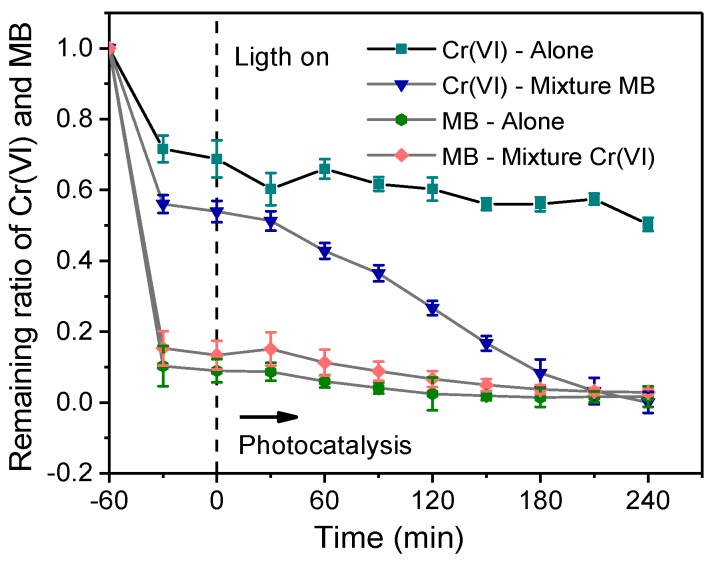
Photocatalysis of Cr(VI) and MB alone or mixed by CNBT under visible light irradiation: *C*_0Cr(VI)_ = 5 mg/L, *C*_0MB_ = 20 mg/L, m/V = 2.5 g/L, pH = 2.0. (−60–0 min: Adsorption process, 0–240 min: Photocatalysis process).

In order to examine possible reactions between Cr(VI) and MB in the studied photocatalytic systems, the removal efficiency of a single pollutant by CNBT was compared with that of the mixed pollutants (the obtained results are shown in Figure 10). It was found that the presence of MB significantly promoted the removal of Cr(VI). When CNBT was used for the photocatalytic treatment of Cr(VI) alone, the removal rate was only about 50%; however, when both pollutants were present in the solution, the Cr(VI) removal rate was 93%. Which means that Cr(VI) in the mixed substrates is higher than that of the single system, the phenomenon is mainly due to the synergistic relationship that takes place between the oxidation of MB and reduction of Cr(VI) onto the surface of the materials, the synergistic effects are benefical to minimize the recombination rate of the charge carriers [51]. As for the influence of Cr(VI) on the MB removal, the adsorption of Cr(VI) was suppressed but the photocatalytic degradation was hardly affected because Cr(VI) competed with MB for the adsorption sites.

#### 3.4.3. Possible Photocatalytic Mechanism in Reaction System

**Figure 11:**
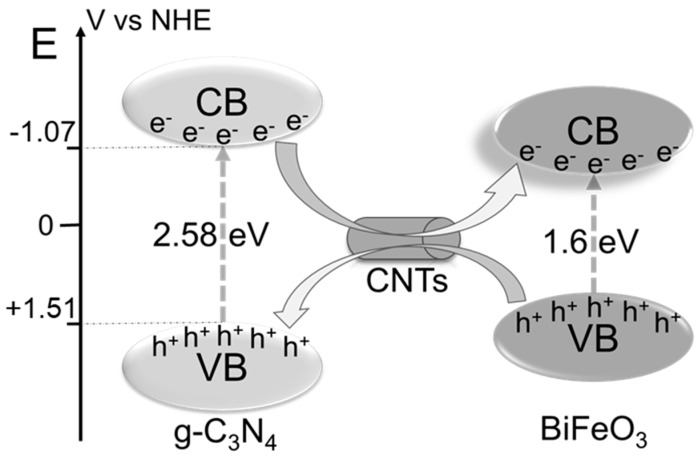
Mechanism diagram of the CNBT composite photocatalytic Cr and MB.

The proposed photocatalytic mechanism of CNBT is shown in Figure 11. When the CNBT photocatalyst is irradiated with visible light that possesses a higher photon energy than its band gap, the catalyst becomes excited to generate a large number of electron-hole pairs [52]. The energy levels of g-C_3_N_4_ was calculated by the Mulliken electronegativity theory [53]:

E_CB_ = *X* − E_e_ − 0.5 E_g_ (3)

E_VB_ = E_CB_ + E_g_ (4)

where *X* is the absolute electronegativity of a semiconductor (X-g-C_3_N_4_ = 4.72 eV); E_e_ is the energy of free electrons compared to hydrogen (4.5 eV) [43]; E_g_ is the band-gap of the g-C_3_N_4_ and BiFeO_3_ are about 2.58 eV and 1.6 eV, respectively. The semiconductor coupling effect between BiFeO_3_ and g-C_3_N_4_ triggers both the electron transfer from the conduction band of g-C_3_N_4_ to that of BiFeO_3_ and hole migration from the valence band of BiFeO_3_ to that of g-C_3_N_4_ [38], while CNTs serve as highly conductive transfer paths that facilitate the migration of these photoexcited charges. In addition, the simultaneous treatment of the organic pollutant MB and heavy metal Cr(VI) can further promote the separation of photoexcited electron-hole pairs. Specifically, photoexcited electrons can react with O_2_ in the photocatalytic system to form the **·**O_2_^−^ species that are able to reduce Cr(VI) [29]. Furthermore, these electrons can also migrate to the material surface and react directly with Cr(VI) [54]. Meanwhile, the photoexcited holes interact with the water adsorbed on the CNBT surface to form the **·**OH radicals, which can subsequently oxidize MB into CO_2_ and H_2_O.

CNBT + *hv* → CNBT (e^−^_CB_ + *h*^+^_VB_) (5)

e^−^ + O_2_ → **·**O_2_^−^ (6)

*h*^+^ + H_2_O → **·**OH + H^+^ (7)

HCrO_4_^−^ + 7H^+^ + 3e^−^ →Cr^3+^ + 4H_2_O (8)

**·**OH + MB → CO_2_ + H_2_O (9)

## 4. Conclusions

In this study, the CNBT ternary magnetic composite photocatalytic material with a high photocatalytic activity was prepared by the hydrothermal synthesis method and then used for the adsorption and photocatalytic treatment of the wastewater containing the heavy metal Cr(VI) and organic dye MB. During the adsorption process, the Cr(VI) and MB species competed for the adsorption sites, but the simultaneous photocatalysis of these two pollutants actually increased their removal rates. In the lower pH range, a stronger Cr(VI) adsorption effect was observed. The obtained equilibrium isotherms were fitted with different two-parameter models, and the Langmuir model produced a better fit than the Freundlich model, suggesting that the distribution of the active sites on the CNBT surface was homogeneous. The results of the kinetic studies showed that the PS-order kinetic model better described the adsorption process and that the adsorption process reached saturation after 0.5 h. The photocatalytic performances of different materials can be ranked in the order of CNBT > BiFeO_3_ > CNB > g-C_3_N_4_. The spectra obtained by UV-Vis, ESR, VSM, PL, and other characterization techniques revealed that the CNBT composite had a reasonable band gap, could be easily separated from the solution, and possessed good visible light response, high chemical stability, and low recombination rates of photoexcited charges, which contributed to its high photocatalytic efficiency. Moreover, photocatalysis likely broke the adsorption-desorption equilibrium achieved over a short time and promoted the deep degradation of pollutants by the studied materials. Therefore, the results obtained in this work can provide a practical and theoretical basis for the treatment of wastewaters containing organic pollutants and heavy metals.

## Supplementary Materials

The following are available online at https://www.mdpi.com/1660-4601/16/17/3219/s1; S1. Adsorption models; S2. Effect of contact time and adsorption kinetics; S3. Adsorption isotherms. Table S1: Kinetic parameters of the Cr(VI) and MB adsorption by CNBT: *C*_0Cr(VI)_ = 5 mg/L, *C*_0MB_ = 20 mg/L, m/V = 2.5 g/L, pH = 3.0, T = 25 °C; Table S2: Comparison of the different isotherm parameters of the Cr(VI) and MB adsorption by CNBT: *C*_0Cr(VI)_ = 5 mg/L, *C*_0MB_ = 20 mg/L, m/V = 2.5 g/L, pH = 3.0, T = 25 °C; Table S3: Magnetic properties of BiFeO_3_, CNB, and CNBT measured. Figure S1: Sorption kinetics data and fitted models of Cr(VI) and MB by CNBT; Figure S2: Different adsorption isotherm models of Cr(VI) (a) and MB (b) by CNBT; Figure S3: Schematic diagram of the photocatalytic activity test reaction scheme. Figure S4: TG-DSC curves of g-C_3_N_4_, BiFeO_3_, CNTs, and CNBT; Figure S5: Hysteresis loops of BiFeO_3_, CNB, and CNBT measured; Figure S6: Comparison of the adsorption effects of g-C_3_N_4_, BiFeO_3_, CNB, and CNBT: *C*_0Cr(VI)_ = 5 mg/L, *C*_0MB_ = 20 mg/L, m/V = 2.5 g/L, pH = 2.0, T = 25 °C.

## References

[1] Han D., Currell M.J., Cao G. (2016). Deep challenges for China’s war on water pollution. Environ. Pollut..

[2] Feng Y., Yang S., Xia L., Wang Z., Suo N., Chen H., Long Y., Zhou B., Yu Y. (2019). In-situ ion exchange electrocatalysis biological coupling (i-IEEBC) for simultaneously enhanced degradation of organic pollutants and heavy metals in electroplating wastewater. J. Hazard. Mater..

[3] Xiao K., He J., Zhang J., Yang B., Zhao X. (2019). Simultaneous Cr(VI) removal and bisphenol A degradation in a solar-driven photocatalytic fuel cell with dopamine modified carbon felt cathode. Appl. Surf. Sci..

[4] Katheresan V., Kansedo J., Lau S.Y. (2018). Efficiency of various recent wastewater dye removal methods: A review. J. Environ. Chem. Eng..

[5] Mitoraj D., Lamdab U., Kangwansupamonkon W., Pacia M., Macyk W., Wetchakun N., Beranek R. (2018). Revisiting the problem of using methylene blue as a model pollutant in photocatalysis: The case of InVO_4_/BiVO_4_ composites. J. Photochem. Photobiol. A Chem..

[6] Zhang Y., Xu M., Li H., Ge H., Bian Z. (2018). The enhanced photoreduction of Cr(VI) to Cr(III) using carbon dots coupled TiO_2_ mesocrystals. Appl. Catal. B Environ..

[7] Ning J., Wang M., Luo X., Hu Q., Hou R., Chen W., Chen D., Wang J., Liu J. (2018). SiO_2_ Stabilized Magnetic Nanoparticles as a Highly Effective Catalyst for the Degradation of Basic Fuchsin in Industrial Dye Wastewaters. Molecules.

[8] Gan L., Zhou F., Owens G., Chen Z. (2018). Burkholderia cepacia immobilized on eucalyptus leaves used to simultaneously remove malachite green (MG) and Cr(VI). Colloids Surf. B Biointerfaces.

[9] Deng Y., Ok Y.S., Mohan D., Pittman C.U., Dou X. (2019). Carbamazepine removal from water by carbon dot-modified magnetic carbon nanotubes. Environ. Res..

[10] Sheng Y., Wei Z., Miao H., Yao W., Li H., Zhu Y. (2019). Enhanced organic pollutant photodegradation via adsorption/photocatalysis synergy using a 3D g-C_3_N_4_/TiO_2_ free-separation photocatalyst. Chem. Eng. J..

[11] Chen F., An W., Liu L., Liang Y., Cui W. (2017). Highly efficient removal of bisphenol A by a three-dimensional graphene hydrogel-AgBr@rGO exhibiting adsorption/photocatalysis synergy. Appl. Catal. B Environ..

[12] Mu C., Zhang Y., Cui W., Liang Y., Zhu Y. (2017). Removal of bisphenol A over a separation free 3D Ag_3_PO_4_ -graphene hydrogel via an adsorption-photocatalysis synergy. Appl. Catal. B Environ..

[13] Cao S., Low J., Yu J., Jaroniec M. (2015). Polymeric Photocatalysts Based on Graphitic Carbon Nitride. Adv. Mater..

[14] Kong L., Mu X., Fan X., Li R., Zhang Y., Song P., Ma F., Sun M. (2018). Site-selected N vacancy of g-C_3_N_4_ for photocatalysis and physical mechanism. Appl. Mater. Today.

[15] Cai X., He J., Chen L., Chen K., Li Y., Zhang K., Jin Z., Liu J., Wang C., Wang X. (2017). A 2D-g-C_3_N_4_ nanosheet as an eco-friendly adsorbent for various environmental pollutants in water. Chemosphere.

[16] Tan J.Z.Y., Nursam N.M., Xia F., Sani M.-A., Li W., Wang X., Caruso R.A. (2017). High-Performance Coral Reef-like Carbon Nitrides: Synthesis and Application in Photocatalysis and Heavy Metal Ion Adsorption. ACS Appl. Mater. Interfaces.

[17] Wu X., Wang X., Wang F., Yu H. (2019). Soluble g-C_3_N_4_ nanosheets: Facile synthesis and application in photocatalytic hydrogen evolution. Appl. Catal. B Environ..

[18] Carvalho K.T.G., Nogueira A.E., Lopes O.F., Byzynski G., Ribeiro C. (2017). Synthesis of g-C_3_N_4_/Nb_2_O_5_ heterostructures and their application in the removal of organic pollutants under visible and ultraviolet irradiation. Ceram. Int..

[19] Zhao H., Yu H., Quan X., Chen S., Zhang Y., Zhao H., Wang H. (2014). Fabrication of atomic single layer graphitic-C_3_N_4_ and its high performance of photocatalytic disinfection under visible light irradiation. Appl. Catal. B Environ..

[20] Wang H.-H., Zhang B., Li X.-H., Antonietti M., Chen J.-S. (2016). Activating Pd nanoparticles on sol-gel prepared porous g-C_3_N_4_/SiO_2_ via enlarging the Schottky barrier for efficient dehydrogenation of formic acid. Inorg. Chem. Front..

[21] Wang X., Yang C., Zhou D., Wang Z., Jin M. (2018). Chemical co-precipitation synthesis and properties of pure-phase BiFeO_3_. Chem. Phys. Lett..

[22] Gao F., Chen X.Y., Yin K.B., Dong S., Ren Z.F., Yuan F., Yu T., Zou Z.G., Liu J.M. (2007). Visible-Light Photocatalytic Properties of Weak Magnetic BiFeO_3_ Nanoparticles. Adv. Mater..

[23] Wang X., Mao W., Zhang J., Han Y., Quan C., Zhang Q., Yang T., Yang J., Li X., Huang W. (2015). Facile fabrication of highly efficient g-C_3_N_4_/BiFeO_3_ nanocomposites with enhanced visible light photocatalytic activities. J. Colloid Interface Sci..

[24] Luo W., Zhu L., Wang N., Tang H., Cao M., She Y. (2010). Efficient Removal of Organic Pollutants with Magnetic Nanoscaled BiFeO_3_ as a Reusable Heterogeneous Fenton-Like Catalyst. Environ. Sci. Technol..

[25] Chen L., Chen N., Wu H., Li W., Fang Z., Xu Z., Qian X. (2018). Flexible design of carbon nanotubes grown on carbon nanofibers by PECVD for enhanced Cr(VI) adsorption capacity. Sep. Purif. Technol..

[26] Hu X., Wang W., Xie G., Wang H., Tan X., Jin Q., Zhou D., Zhao Y. (2019). Ternary assembly of g-C_3_N_4_/graphene oxide sheets /BiFeO_3_ heterojunction with enhanced photoreduction of Cr(VI) under visible-light irradiation. Chemosphere.

[27] Noimark S., Weiner J., Noor N., Allan E., Williams C.K., Shaffer M.S.P., Parkin I.P. (2015). Dual-Mechanism Antimicrobial Polymer-ZnO Nanoparticle and Crystal Violet-Encapsulated Silicone. Adv. Funct. Mater..

[28] Deng Y., Tang L., Zeng G., Feng C., Dong H., Wang J., Feng H., Liu Y., Zhou Y., Pang Y. (2017). Plasmonic resonance excited dual Z-scheme BiVO_4_/Ag/Cu_2_O nanocomposite: Synthesis and mechanism for enhanced photocatalytic performance in recalcitrant antibiotic degradation. Environ. Sci. Nano.

[29] Anirudhan T.S., Shainy F., Christa J. (2017). Synthesis and characterization of polyacrylic acid- grafted-carboxylic graphene/titanium nanotube composite for the effective removal of enrofloxacin from aqueous solutions: Adsorption and photocatalytic degradation studies. J. Hazard. Mater..

[30] Tseng W.J., Lin R.D. (2014). BiFeO_3_/alpha-Fe_2_O_3_ core/shell composite particles for fast and selective removal of methyl orange dye in water. J. Colloid Interface Sci..

[31] Sankar Ganesh R., Sharma S.K., Sankar S., Divyapriya B., Durgadevi E., Raji P., Ponnusamy S., Muthamizhchelvan C., Hayakawa Y., Kim D.Y. (2017). Microstructure, structural, optical and piezoelectric properties of BiFeO_3_ nanopowder synthesized from sol-gel. Curr. Appl. Phys..

[32] Kang H.W., Lim S.N., Song D., Park S.B. (2012). Organic-inorganic composite of g-C_3_N_4_-SrTiO_3_:Rh photocatalyst for improved H_2_ evolution under visible light irradiation. Int. J. Hydrog. Energy.

[33] Cheng N., Tian J., Liu Q., Ge C., Qusti A.H., Asiri A.M., Al-Youbi A.O., Sun X. (2013). Au-Nanoparticle-Loaded Graphitic Carbon Nitride Nanosheets: Green Photocatalytic Synthesis and Application toward the Degradation of Organic Pollutants. ACS Appl. Mater. Interfaces.

[34] Chang Y.P., Ren C.L., Qu J.C., Chen X.G. (2012). Preparation and characterization of Fe_3_O_4_/graphene nanocomposite and investigation of its adsorption performance for aniline and p-chloroaniline. Appl. Surf. Sci..

[35] Hu X.J., Liu Y.G., Zeng G.M., Wang H., Hu X., Chen A.W., Wang Y.Q., Guo Y.M., Li T.T., Zhou L. (2014). Effect of aniline on cadmium adsorption by sulfanilic acid-grafted magnetic graphene oxide sheets. J. Colloid Interface Sci..

[36] Zhou C., Lai C., Huang D., Zeng G., Zhang C., Cheng M., Hu L., Wan J., Xiong W., Wen M. (2018). Highly porous carbon nitride by supramolecular preassembly of monomers for photocatalytic removal of sulfamethazine under visible light driven. Appl. Catal. B Environ..

[37] Yang M.Q., Zhang N., Xu Y.J. (2013). Synthesis of fullerene, carbon nanotube, and graphene-TiO_2_ nanocomposite photocatalysts for selective oxidation: A comparative study. ACS Appl. Mater. Interfaces.

[38] An J., Zhang G., Zheng R., Wang P. (2016). Removing lignin model pollutants with BiFeO_3_/g-C_3_N_4_ compound as an efficient visible-light-heterogeneous Fenton-like catalyst. J. Environ. Sci..

[39] Wang X., Lu M., Ma J., Ning P., Che L. (2018). Synthesis of K-doped g-C_3_N_4_/carbon microsphere@graphene composite with high surface area for enhanced adsorption and visible photocatalytic degradation of tetracycline. J. Taiwan Inst. Chem. Eng..

[40] Di J., Li S., Zhao Z., Huang Y., Jia Y., Zheng H. (2015). Biomimetic CNT@TiO_2_ composite with enhanced photocatalytic properties. Chem. Eng. J..

[41] Li S., Li Z., Ke B., He Z., Cui Y., Pan Z., Li D., Huang S., Lai C., Su J. (2019). Magnetic multi-walled carbon nanotubes modified with polyaluminium chloride for removal of humic acid from aqueous solution. J. Mol. Liq..

[42] Booshehri A.Y., Chun-Kiat Goh S., Hong J., Jiang R., Xu R. (2014). Effect of depositing silver nanoparticles on BiVO_4_ in enhancing visible light photocatalytic inactivation of bacteria in water. J. Mater. Chem. A.

[43] Bai X., Du Y., Hu X., He Y., He C., Liu E., Fan J. (2018). Synergy removal of Cr (VI) and organic pollutants over RP-MoS_2_/rGO photocatalyst. Appl. Catal. B Environ..

[44] Ning J., He Q., Luo X., Wang M., Liu D., Wang J., Li G., Liu J. (2018). Determination of Uric Acid in Co-Presence of Dopamine and Ascorbic Acid Using Cuprous Oxide Nanoparticle-Functionalized Graphene Decorated Glassy Carbon Electrode. Catalysts.

[45] Zhang Y., Zhang Q., Shi Q., Cai Z., Yang Z. (2015). Acid-treated g-C_3_N_4_ with improved photocatalytic performance in the reduction of aqueous Cr(VI) under visible-light. Sep. Purif. Technol..

[46] Liu W., Cao L., Cheng W., Cao Y., Liu X., Zhang W., Mou X., Jin L., Zheng X., Che W. (2017). Single-site active cobalt-based photocatalyst with long carriers lifetime for spontaneous overall water splitting. Angew. Chem. Int. Ed..

[47] Liu W., Wang M., Xu C., Chen S. (2012). Facile synthesis of g-C_3_N_4_/ZnO composite with enhanced visible light photooxidation and photoreduction properties. Chem. Eng. J..

[48] Qin F., Wang R., Li G., Tian F., Zhao H., Chen R. (2013). Highly efficient photocatalytic reduction of Cr(VI) by bismuth hollow nanospheres. Catal. Commun..

[49] Wang H., Yuan X., Wang H., Chen X., Wu Z., Jiang L., Xiong W., Zeng G. (2016). Facile synthesis of Sb_2_S_3_/ultrathin g-C_3_N_4_ sheets heterostructures embedded with g-C_3_N_4_ quantum dots with enhanced NIR-light photocatalytic performance. Appl. Catal. B Environ..

[50] Ibrahim S., Shuy W.Z., Ang H.-M., Wang S. (2010). Preparation of bioadsorbents for effective adsorption of a reactive dye in aqueous solution. Asia Pac. J. Chem. Eng..

[51] Alam U., Khan A., Bahnemann D., Muneer M. (2018). Synthesis of Co doped ZnWO_4_ for simultaneous oxidation of RhB and reduction of Cr(VI) under UV-light irradiation. J. Environ. Chem. Eng..

[52] Tang L., Feng C., Deng Y., Zeng G., Wang J., Liu Y., Feng H., Wang J. (2018). Enhanced photocatalytic activity of ternary Ag/g-C_3_N_4_/NaTaO_3_ photocatalysts under wide spectrum light radiation: The high potential band protection mechanism. Appl. Catal. B Environ..

[53] Zhang D., Cui S., Yang J. (2017). Preparation of Ag_2_O/g-C_3_N_4_/Fe_3_O_4_ composites and the application in the photocatalytic degradation of Rhodamine B under visible light. J. Alloy. Compd..

[54] Zhang Y., Ma H.-L., Peng J., Zhai M., Yu Z.Z. (2012). Cr(VI) removal from aqueous solution using chemically reduced and functionalized graphene oxide. J. Mater. Sci..

