# Supplementary material for: Synergy of Photocatalysis and Adsorption for Simultaneous Removal of Hexavalent Chromium and Methylene Blue by g-C3N4/BiFeO3/Carbon Nanotubes Ternary Composites
Source: Int J Environ Res Public Health. 2019 Sep 3;16(17):3219. doi: 10.3390/ijerph16173219 (PMC6747399; doi:10.3390/ijerph16173219)
Supplement: Supplementary file 1 [file ijerph-16-03219-s001.pdf]

International Journal of
Environmental Research and Public Health

MDPI

Supplementary Material for

Synergy of photocatalysis and adsorption for simultaneous removal of hexavalent chromium and methylene blue by g-C_3N_4/$BiFeO_3$/carbon nanotubes ternary composites

Huiwen Huo [1], Xinjiang Hu [1,*], Hui Wang [1,2], Jiang Li [3], Guangyu Xie [1], Xiaofei Tan [4,5], Qi Jin [1], Daixi Zhou [1], Chuang Li [1], Guoqiang Qiu[2], Yunguo Liu [4,5]

[1] College of Environmental Science and Engineering, Central South University of Forestry and Technology, Changsha 410004, P.R. China; HHWens@126.com (H.H.); wanghui@csuft.edu.cn (H.W.); anyuxie@126.com (G.X.); jq305745239@126.com (Q. J.); daixizhou07@126.com (D.Z.); lc302864860@163.com (C. L.)
[2] Faculty of Life Science and Technology, Central South University of Forestry and Technology, Changsha 410004, P.R. China; qiuguoqiang1995@163.com (G.Q.)
[3] School of Architecture and Art, Central South University, Changsha 410082, P.R. China; lijiang@csu.edu.cn; lijiang@csu.edu.cn
[4] College of Environmental Science and Engineering, Hunan University, Changsha 410082, P.R. China; tanxf@hnu.edu.cn (X.T.); hnuese@126.com (Y.L.)
[5] Key Laboratory of Environmental Biology and Pollution Control (Hunan University), Ministry of Education, Changsha 410082, P.R. China
[*] Corresponding author: huxinjiang@126.com, xjhu@csuft.edu.cn (X.H.); Tel.: 0731-85623096 (X.H.)

International Journal of
Environmental Research and Public Health

S1. Adsorption models

To evaluate the adsorption efficiency of the adsorbent, the pseudo-first-order (PF-order) and pseudo-second-order (PS-order) adsorption kinetic models were considered. Their corresponding fitting equations are as follows:

$$q_t = q_e(1-e^{-k_1 t}) \quad (1)$$

PS-order kinetics equation:

$$q_t = \frac{q_e^2 k_2 t}{1 + q_e k_2 t} \quad (2)$$

where q_t (mg/g) is the amount of the adsorbed material at time t (min); q_e is the adsorbed amount at equilibrium (mg/g); and k_1 and k_2 are the adsorption rate constants of the PF-order (min^{-1}) and PS-order (min^{-1}) models, respectively [1].

To determine the adsorption efficiency, the obtained experimental data were simulated using the Langmuir and Freundlich isotherm models. The former model assumes the formation of a monolayer after adsorption saturation, and the latter model assumes that a multi-molecular layer with different energies of various adsorption sites is formed. The equation for the Langmuir model can be written as:

$$q_e = \frac{q_m K_L C_e}{1 + K_L C_e} \quad (3)$$

where K_L is the Langmuir adsorption constant (L/mg), C_e is the equilibrium amount of the adsorbed material (mg/L), and q_m is the adsorbed amount (maximum adsorption capacity) corresponding to the formation of a complete monolayer on the adsorbent surface (mg/g).

The equation for the Freundlich isotherm model can be expressed as follows:

$$q_e = K_F C_e^{\frac{1}{n}} \quad (4)$$

Here, K_F and n are the Freundlich model constants that are related to the adsorption capacity and adsorption strength, respectively [2].

S2. Effect of contact time and adsorption kinetics

Adsorption kinetics may not only help to elucidate the adsorption mechanism, but also evaluate the performance of the adsorbent. The measured adsorption capacities of CNBT were fitted and analyzed by the PF-order and PS-order kinetic models, as shown in Figure S1. The adsorption of Cr(VI) and MB species reached equilibrium within the first 0.5 h, and their removal rates were 57% and 80%, respectively. Afterwards, no significant adsorption of Cr(VI) was observed in the next 4.5 h, and weak desorption of MB was detected. The initial rapid adsorption may be attributed to the presence of a large number of Cr(VI) and MB species on the adsorbent surface, while the late adsorption stage was relatively slow because of the saturation of the adsorption sites [3]. Owing to the limited number of adsorption sites on the CNBT surface, the competition between Cr(VI) and MB species for these sites may also lead to the weak desorption of MB.

Table S1 lists the fitting parameters obtained for the CNBT composite. They show that the adsorption of Cr(VI) and MB can be described by both the first-order and second-order kinetic models; however, more accurate fitting was achieved for the second-order model indicating that the

adsorption rate linearly increased with the concentrations of Cr(VI) and MB, suggesting the overall adsorption process was controlled by chemisorption. Besides, the adsorption process was dependent on the number of available adsorption sites on the surface of the adsorbent and eventually controlled by the attachment of the adsorbates on the surface[4]. Besides, according to Table S1, the MB adsorption rate was lower than that of Cr(VI).

S3. Adsorption isotherms

The fitting results obtained for the Langmuir and Freundlich models are presented in Table S2 and Figure S2, respectively. Langmuir model normally describes the adsorption isotherm of adsorbate on completely homogeneous surface of adsorbent, Freundlich model is commonly used for the description of multilayer adsorption of adsorbates on heterogeneous adsorbent surface[4]. They show that the adsorption capacities of Cr(VI) and MB increased with increases in their initial concentrations and ultimately reached equilibria. As the concentrations of Cr(VI) and MB increased, the adsorption sites became gradually occupied and finally reached saturation [1]. The obtained correlation coefficient ($R^2 \geq 0.9$) listed in Table S2 indicates that the adsorption process is more consistent with the Langmuir model and that the Cr(VI) and MB adsorption sites are homogeneously distributed on the CNBT surface, which lead to monolayer adsorption and there is no interaction between the adsorbate molecules. [5]. Therefore, the adsorption capacity is proportional to both the specific surface area of the adsorbent and number of adsorption sites.

S4. Supplementary Tables

Table S1. Kinetic parameters of Cr(VI) and MB adsorption by CNBT: $C_{0Cr(VI)}$=5 mg/L, C_{0MB}=20 mg/L, m/V=2.5 g/L, pH=3.0, T=25 °C.

Pollutants	Pseudo-first-order-kinetics			Pseudo-second-order-kinetics		
	q_e (mg/g)	k_1 (min^{-1})	R^2	q_e (mg/g)	k_2 (min^{-1})	R^2
Cr(VI)	1.19±0.02	0.39±0.06	0.983	1.21±0.02	0.98±0.03	0.986
MB	6.07±0.04	0.40±0.03	0.995	6.15±0.05	0.22±0.04	0.996

Table S2. Comparison of different isotherm parameters of Cr(VI) and MB adsorption by CNBT: $C_{0Cr(VI)}$=5 mg/L, C_{0MB}=20 mg/L, m/V=2.5 g/L, pH=3.0, T=25 °C.

Models	Langmuir model			Freundlich model		
	q_m (mg/g)	K_L (L/mg)	R^2	n	K_F (mg/g)	R^2
Cr(VI)	1.10±0.05	5.14±1.69	0.900	7.13±1.23	0.75±0.05	0.897
MB	17.72±1.50	1.16±0.42	0.911	4.92±1.26	6.91±1.53	0.814

Table S3. Magnetic properties of $BiFeO_3$, CNB and CNBT measured.

	Ms (emu/g)	Mr (emu/g)	Hc (Oe)
$BiFeO_3$	2.95	0.29	75.99
CNB	1.85	0.14	56.76
CNBT	1.30	0.12	69.69

S5. Supplementary Figures

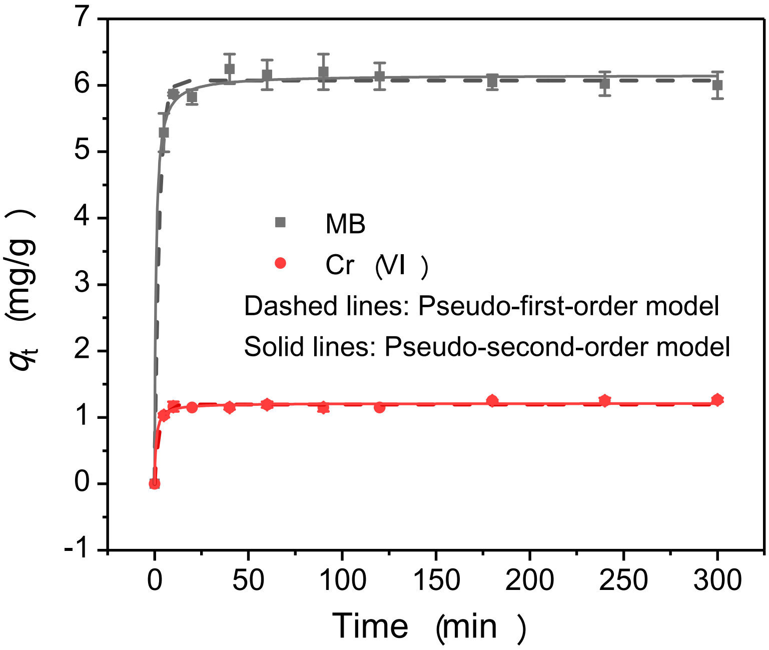

Figure S1. Sorption kinetics data and fitted models of Cr (VI) and MB by CNBT.

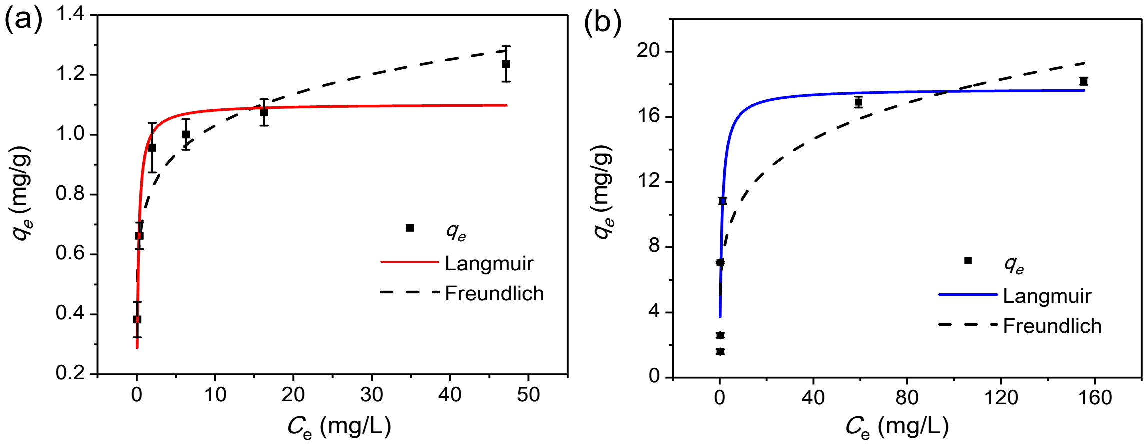

Figure S2. Different adsorption isotherm models of Cr(VI) (a) and MB (b) onto by CNBT.

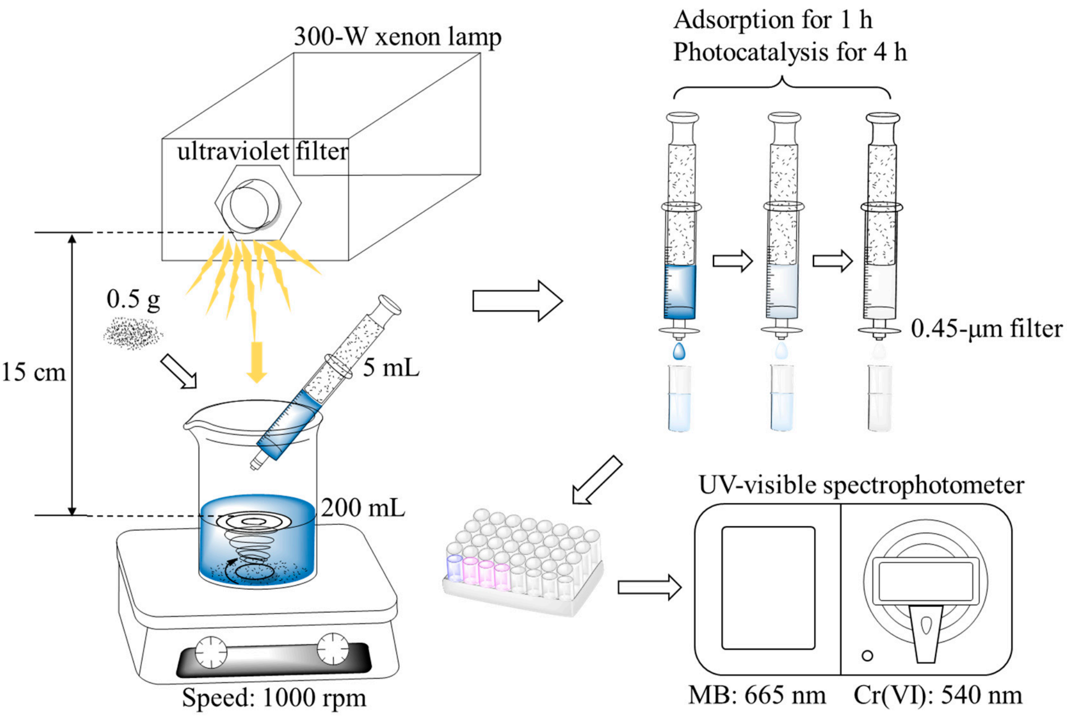

Figure S3. Schematic diagram of photocatalytic activity test reaction scheme.

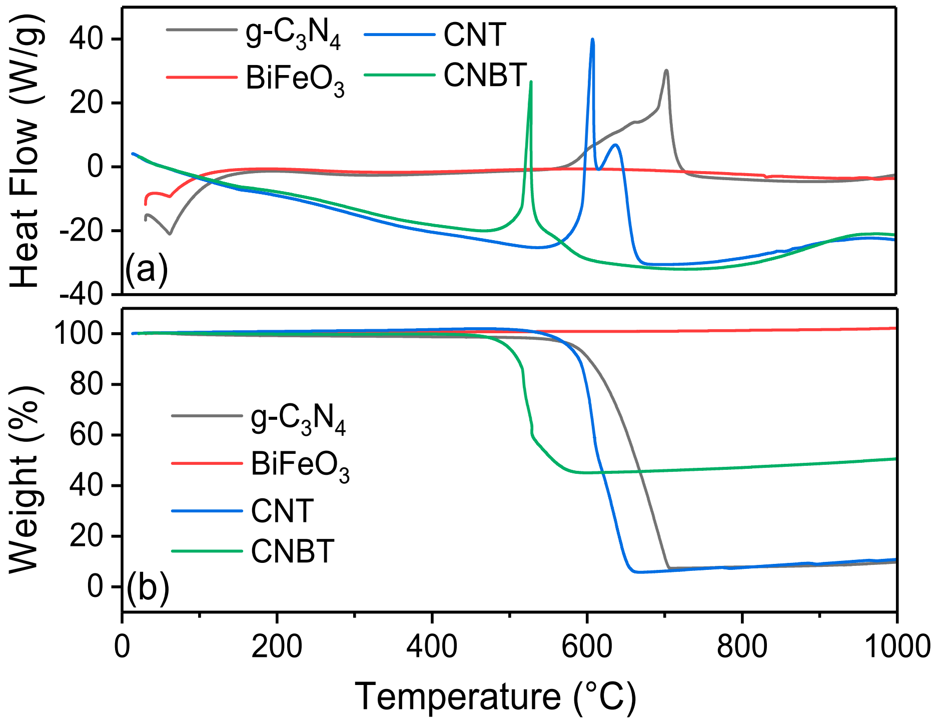

Figure S4. TG-DSC curves of g-C_3N_4, $BiFeO_3$, CNTs and CNBT.

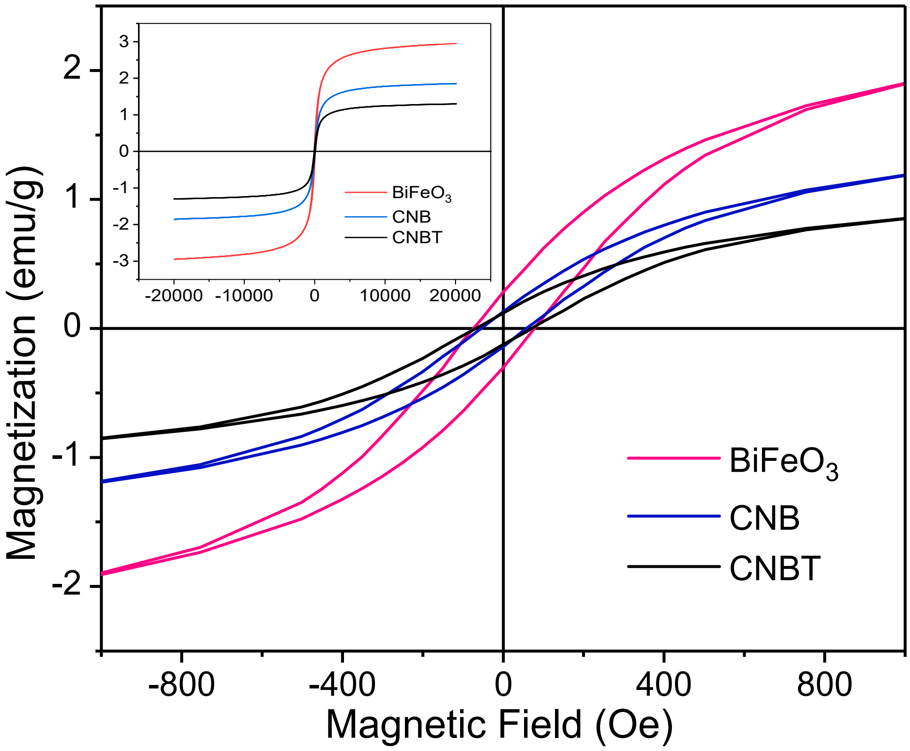

Figure S5. Hysteresis loops of $BiFeO_3$, CNB and CNBT measured.

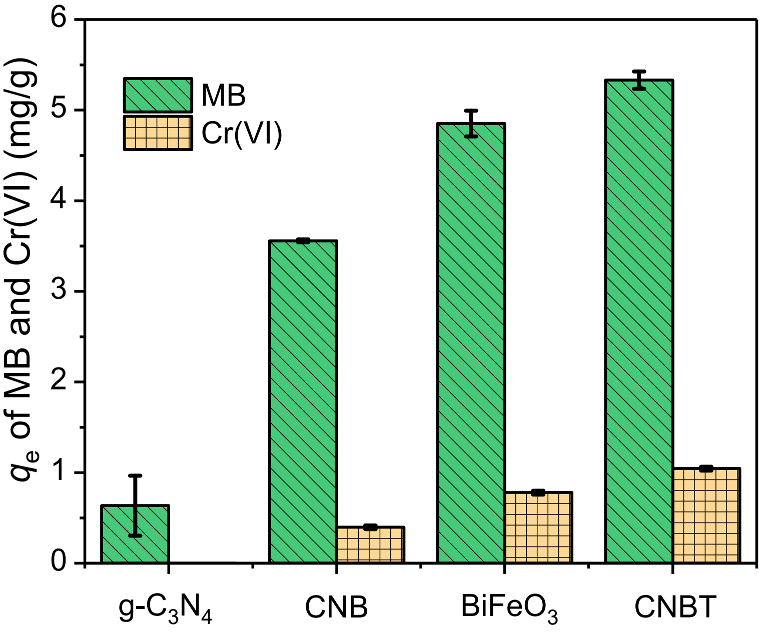

Figure S6. Comparison of adsorption effects of g-C_3N_4, $BiFeO_3$, CNB and CNBT: $C_{0Cr(VI)}$=5 mg/L, C_{0MB}=20 mg/L, *m*/*V*=2.5 g/L, pH=2.0, *T*=25 °C.

References

1. Chen, Y.; Wang, B.; Xin, J.; Sun, P.; Wu, D., Adsorption behavior and mechanism of Cr(VI) by modified biochar derived from Enteromorpha prolifera. *Ecotoxicology And Environmental Safety* **2018**, *164*, 440-447.

2. He, X.; Male, K.B.; Nesterenko, P.N.; Brabazon, D.; Paull, B.; Luong, J.H., Adsorption and desorption of methylene blue on porous carbon monoliths and nanocrystalline cellulose. *Acs Applied Materials & Interfaces* **2013**, *5*, 8796-8804.
3. Chang, Y.-P.; Ren, C.-L.; Qu, J.-C.; Chen, X.-G., Preparation and characterization of Fe_3O_4/graphene nanocomposite and investigation of its adsorption performance for aniline and p-chloroaniline. *Applied Surface Science* **2012**, *261*, 504-509.
4. Min, L.-L.; Yuan, Z.-H.; Zhong, L.-B.; Liu, Q.; Wu, R.-X.; Zheng, Y.-M., Preparation of chitosan based electrospun nanofiber membrane and its adsorptive removal of arsenate from aqueous solution. *Chemical Engineering Journal* **2015**, *267*, 132-141.
5. Zheng, Y.M.; Yu, L.; Chen, J.P., Removal of methylated arsenic using a nanostructured zirconia-based sorbent: process performance and adsorption chemistry. *Journal Of Colloid And Interface Science* **2012**, *367*, 362-369.
